# The Speculum Applied to the Diagnostic and Treatment of the Organic Diseases of the Womb. An Inaugural Dissertation, &c.

**Published:** 1837-01-01

**Authors:** 


### THE SPECULUM APPLIED TO THE DIAGNOSTIC AND TREATMENT OF THE ORGANIC DISEASES OF THE WOMB. An Inaugural Dissertation, presented to the University of Glasgow, for the Degree of Doctor in Medicine. By *John Balbirnie*, A.M. Octavo, pp. 335. Longman, 1836.

THERE can be no doubt of the propriety, indeed necessity, of rendering medicine as strictly a science of induction as possible. The more exact it is the better. If Lord Bacon were to revisit us for a brief period, and to observe the alteration that has occurred in physic, since, in his "advancement of human learning," he discoursed on its condition in his own day, he would be astonished at alterations, which himself, the apostle of induction, has originated. Mead and Heberden, the ornaments of the old school, no longer represent the new. The foundation of their art was empirical observation. Such observation could never lead to any stable results, because its subjects were phenomena, themselves not simple, nor dependent on a single cause. Their diagnoses, prognoses, hypotheses, and modes of treatment were essentially derived from the study of symptoms. But as whole groups of symptoms are common to many different physical alterations, the investigation of them unconnected with an acquaintance with those alterations too, must continually perplex and frequently deceive. Hippocrates had genius enough to see the difficulties of his system, and the ars longa, observatio difficilis, experientia fallax of his aphorism, are the honest confessions of its peculiar imperfection.

The great instrument of improvement to modern medicine has been morbid anatomy. The benefits it has conferred are both positive and negative —positive, where it has shewn the physical changes which constitute some diseases—negative, where it has proved that no physical changes are essential to others.

In proportion as the alterations which constitute disease are capable of being appreciated by the senses, our notions of that particular malady are more determinate and satisfactory. This is the advantage which surgery possesses over physic. The subjects of the former are palpable and immediate—those of the latter more shadowy and remote. And the consequence is, that surgeons are frequently more exact in their ideas of disease than physicians are.

The more we can render symptoms objects of sense, the less likely we are to mistake them. This is so obvious as to constitute a truism. Yet such a truism was a subject of ridicule when Laennec first advocated auscultation. So obstinate is prejudice, so blind is error. Is there now a man in the profession who would publicly ridicule the principles of auscultation? Not one. Auscultation does no more than tend to render us better acquainted with the physical alterations of the lungs, by making their physical effects sensible. How absurd must they have been who could think this absurd.

The diseases of the uterus are not few, nor trivial. They affect a sex whose character and weakness claim our instinctive sympathy, and demand all our assistance. That sex is peculiarly circumstanced. It owes much of the respect that it receives to its exquisite perception of modesty and delicacy. Those feminine attributes are fostered and increased by the homage which men pay them, a homage the result of a high state of civilization, and the intellectual descendant of the grosser courtesies of the age of chivalry. To their sense of modesty, carried to excess, women chiefly owe their present high position in civilized society; and if it shall be found that some evils spring from it, they are probably only the customary tax which is paid for whatever is useful and precious.

Nature and reason, instinct and reflection, contribute to surround the generative organs and functions of women with mystery to themselves. Modesty is so wrought up with an apparent ignorance respecting their uses and their states, and with an aversion to all investigations into either, that we think even a whore shameless who does not evince a repugnance to the display of them. In the eye of philosophy this appears ridiculous—in the code of cold utilitarianism it may seem mischievous—but when the good and the evil that spring from it are balanced—when what woman gets by her modesty is set against all she loses by it, the advantage will probably be found on its side.

The study of diseases of the uterus is therefore a peculiar case, and it is not clear that we can apply to it the same principles which we employ in the investigation of the diseases of other organs. We can have no hesitation in endeavouring to ascertain the state of a female's lungs, by placing a wooden tube upon the chest, because this may be done without calling any blush, save that of trepidation, to the most modest cheek. But it is very doubtful what amount of good can compensate a young girl, or even a wife, for being placed upon a bed or table, her thighs held wide asunder, a speculum thrust into her vagina, and screwed open till all within is exposed, while a man is curiously looking in, by the light of the sun or of a tallow candle.

Many of us know how delicately they manage all this in France. Dr. Balbirnie, the avowed "apostle" of the speculum, is fresh from that country, where he saw it employed upon a noble scale. M. Lisfranc has his appointed days for a "grande revue de speculum." Followed by a long array of young and laughing students, he enters the ward where the females are to undergo this chaste inspection. Each woman is on the bed, with her knees drawn up, and her thighs wide apart—the speculum is in. M. Lisfranc looks first. Dieu! que c'est magnifique! The students follow, and as the candle passes from one to the other, each looks intently up the vagina.

Impressed with the advantages of such inspections, Dr. Balbirnie advocates the use of the speculum extensively in this country, with singular earnestness and zeal. He sets out "upon the broad and irrefragable grounds,—

"1st. *That it is the imperative duty of the medical practitioner—a duty, which the best interests of humanity, as well as the obligations of his professional vow, imposes upon him—to arm himself with every means that can afford him more precise knowledge of the seat and nature of disease; and, consequently, render more rational and more certain his mode of treatment.* There is a path of duty plainly and palpably set before him, which he cannot flinch from. And he must set himself boldly, but delicately, to oppose the very hurtful, though very pardonable, prejudices, that interfere with the discharge of this duty, and that tend to perpetuate the evils that are the lot of the fairest portion of humanity.

2d. *That the practitioner is not competent to give advice on the diseases of the womb particularly, nor to undertake their treatment, till, by a speculum-examination, he has fully satisfied himself of the pathological condition of the organ he has to do with.* This will appeal to the good sense of every man, as the only method that will produce results, important both for science and for humanity.

We know well the tenacity in the minds of men of long-cherished prejudices, and old-received opinions. We shall be called by many, doubtless, an *innovator*. But we have the consolation to think, that all the best contributions presented by science to modern medicine, have met with opposition; ay, and virulent opposition sometimes; and their advocates have been styled *innovators*. What opposition did not Harvey's discovery of the circulation of the blood create! He, also, was an *innovator*. How was the introduction of the vaccine inoculation first received? Jenner, also, was an *innovator*. The science of auscultation, also, was once opposed in Great Britain; and is, perhaps, still opposed by the physicians of the 'ancien régime.' But all these have long achieved their triumph, and each created a new era in the history of medicine. Doubtless, a similar opposition awaits the speculum; but we hope for it ultimately a similar triumph, and that it will create for itself a similar era. We go forth its advocate. We proclaim ourselves, '*the apostle of the speculum*.' With that instrument we link our fate; and by that we will stand or fall." 14.

This is very heroic. All hail, Balbirnie, apostle of the speculum! A holy mission. We may imagine the zealous saint, eager to enter on his ministry, sallying forth and brandishing his peculiar weapon. We may conceive him addressing a crowd of ladies, and earnestly entreating them to allow him to experiment on each and all with the most religious sanctity and secrecy. He would implore them to open their "sanctum sanctorum," with such protestations as these.

"We hope, that so far as regards ourselves, all that dignity and that delicacy of feeling which ought to distinguish the medical character, will be for us a solemn duty, and a solemn observance, never to be infringed, never to be compromised. If at any time the medicel character should merge into, and invest itself with all the sanctity of the sacerdotal, it is certainly in such circumstances. He is for the time a high-priest—the alone privileged to enter into a *sanctum sanctorum*, whose recesses are not to be exposed to vulgar gaze, as its revelations are to remain buried in the depths of his own heart." 20.

Could the ladies resist such a moving appeal—could they remain obdurate while the Doctor prayed and screwed and unscrewed his favourite implement? He would undoubtedly prevail, for he is convinced and he assures us that:—

"The difficulties on this score have been magnified by practitioners. If the physician is firm in insisting on the necessity of such examination, and if he knows how to do his duty in this way properly, shewing a corresponding personal delicacy, and sympathy for their situation, the very pardonable scruples of the patient will give way to her good sense, and the natural timidity of the sex will yield to the sentiment of self-preservation. The ladies are not obdurate. Their character is to be 'gentle and easily entreated.' They will be reasoned with, and submit to what is reasonable. When it is their own best interests that are at stake, they will see the prudence and propriety of a timely submission to every means necessary for their cure: and the results of the investigation—the satisfaction and assurance of their own mind as to the nature of the disease, and a rational and successful treatment founded on it, will be to them an ample recompense for the painful sacrifice of their delicacy it for the moment exacted." 20.

It must be owned that the Doctor is somewhat empressé, and that he has an itching palm for pulling up the ladies' petticoats. It remains to be seen whether they will be so easily entreated as their apostle appears to believe. That is a matter for him and for them to settle between them. Dr. Balbirnie's new-born ardor reminds us of what Sheridan said of one of his countrymen, Lord Glenbervie. His Lordship, a highlander, had always worn kilts until his appearance in the House of Commons. He entered it arrayed in a large pair of breeches. "Look at Glenbervie," said one of the members; "did ever man wear such enormous breeches." "Ah," cried Sheridan, "converts, you know, are always enthusiasts."

When we examine the reasons that may be urged in favour of the speculum, we shall probably arrive at some such conclusions as these.

Its employment may be painful, and is disgusting. It is highly disagreeable to the surgeon or physician, and must be revolting to every woman of delicacy. Strong reasons only, in any particular case, can justify its use upon modest females.

The characters of the discharges and the general symptoms, will usually enable us to determine whether the uterus is affected. When they are insufficient, we possess a means of physical examination which, in the great majority of cases, is quite as satisfactory as the employment of the speculum, and possesses not the hundredth part of its indecency. We mean, of course, examination by the finger. One would suppose, from Dr. Balbirnie's florid enumeration of the benefits arising from the use of the speculum, that it was impossible to distinguish by the touch chronic enlargement of the neck of the uterus, or ulcerations upon it. The fact is that most of the diseases of the neck are detected without much difficulty in this manner. If uterine diseases are often overlooked, it is not because we want the means of ascertaining them, but because the modesty of patients prevents our resorting to them.

In other words our ordinary mode of diagnosis is little used because indecent, while, compared with the speculum, it is modesty itself.

When we set against that instrument these objections—that its employment is disgusting—that it helps us little, or not at all in the most obscure cases, those in which the body or fundus of the uterus is the part affected——and, that in the great majority of instances, other means of diagnosis are sufficient for ordinary practical purposes—we seal the fate of the speculum, as an instrument of common application. We venture to prophecy that it never will come into *general* use in this country. Nay, we will go so far as to observe that it is right it never should. Not but that additional exactness is an excellent thing; only, in this case it would be bought too dear. The fact is that there are two parties to the bargain—the doctors and the ladies. If the former were ever so inclined to use the speculum, the latter may, and they will reject it.

The cases in which there are the least moral objections to the instrument, are those of a venereal character. If a woman has been immodest enough to be poxed, she may very well afford to submit to the introduction of the speculum. Yet the surgeon will be disappointed, if he anticipates *often* finding syphilitic ulcerations deep in the vagina. We regularly examined for some months with the speculum, the women admitted into the Lock Hospital. In only two or three instances did we find deep ulcerations, and, in each of those, if our memory serves us, there were sores on the external parts. The only interesting fact that we elicited was the frequency of uterine discharges.

After the eloquent advocacy of the speculum, to which we have sufficiently alluded, our author presents us with a "brief historical sketch of the diseases of the womb, from the father of medicine downwards." The heading is ambiguous, and, at first glance, reads, as if Dr. Balbirnie began with the diseases of the womb of the father of medicine. This, however, is not the case, for it is upon the doctrines of Hippocrates, and not upon his womb, that our author descants. The sketch of the opinions of the ancient doctors is amusing and not useless, but would of course be out of place here. We shall only extract one passage, alluding to by-gone notions of hysteria, which, although familiar to many of our readers, will probably be new to others. The ancients, it must be premised, regarded the uterus as almost a separate being, an animal in animale. They were sorely puzzled what to think of it in cases of hysteria,

"The symptoms which constitute this state, were attributed, as every one knows, to the circumstance that the womb, capable of locomotion in the abdomen, was transported sometimes to the right, sometimes to the left, sometimes ascending above, at other times shifting below. Now, it was to the movement of ascension, and we know wherefore, that the severest accidents were attributed. And because disagreeable odours appeared to exercise a special salutary influence on women in this state (any thing, in fact, which produces a lively impression, has the same effect), it was concluded that the womb avoided disagreeable odours, and that, on the contrary, it sought perfumes. Hence the practice, as absurd as indecent, of offering it perfumes, by means of aromatic pessaries, or fumigations, carried into the vagina by instruments made express. These ideas Moschion blames; and he is the only author who, until a very recent epoch, has talked with so much good sense on the subject. For Chambon, indeed, whose work was printed in the year VII. of the French republic, recommends still these means, and repeats with the ancients, that it is a mode of distinguishing the suffocation of the womb from other diseases which have some resemblance to it." 33.

The first chapter, for we now fairly enter on the work, is on the uterus and its exploration. An anatomical description of the organ is presented. It might have been judiciously withheld, for there is in it nothing new, and our author merely copies from the systems of anatomy.

But the mode of exploration with the speculum is more deserving of attention. Many, perhaps, of our country readers may not feel quite at home in this, and as the speculum will probably be employed more than it has been, we think it may be well to extract the directions of Dr. Balbirnie. He is speaking of the two-branched instrument of M. Ricord.

"The patient is placed upon the side of a bed, a pillow under the shoulders, and under the head, the thighs semi-flexed upon the pelvis, the feet resting on chairs, placed at either side. The practitioner stands between, and has no need of an assistant; a thing very important in private practice. The speculum, slightly warmed, if it is cold weather, is anointed with an oily substance, to permit the instrument to slide more smoothly, and with less pain; and which does not alter the appearance of the secretions, nor of the tissues, to be observed. The valves of the speculum, held in the right hand, are firmly compressed. Separating, then, the great and the small labia with the ring and index finger of the left hand, the fourchette, and the posterior part of the vaginal ring, are, at the same time, depressed with the middle finger of the same hand. This manœuvre, very important for facilitating, without pain, the entry of the speculum, ought to be made in a gradual manner, but sufficiently strong. Then, the extremity of the speculum is presented to the vagina, its branches turned towards the left thigh; whilst the edge of the extremity of one of the valves is placed strongly against the left middle finger; placed, as we have said, the plain surface of the other branch is applied against the posterior face of the prominence of the meatus urinarius, beneath which it is made to slide by a rapid motion, without excoriating or injuring this part, as happens frequently by the other methods. As soon as the exterior opening is cleared, which is the most difficult and painful part of the process, the instrument is guided in the direction of the known axis of the vagina, its valves being more or less separated, to permit us to explore, successively, the whole length of the vagina, before it goes to embrace the neck of the uterus. The position and height of the os tincæ being ascertained by the previous touch, the extremity of the instrument is directed accordingly, recommending to the patient, to make no expulsive efforts, which would obstruct the progress of the instrument. In thus gently separating, with the end of the instrument, the upper and posterior walls of the vagina, we soon reach the neck, which is recognised by its smooth polished mucus, and by its tint, differing generally from that of the vagina. In some cases, the glairy mucosities, exuding from its orifice, and descending in the vagina, indicate the route to follow. If, in spite of these indications and precepts, the end of the speculum is engaged in the *cul de sac* of the vagina, instead of continuing to push, a slight movement of retreat, in separating the valves, generally succeeds in seizing the os tincæ.

The speculum, introduced, is kept in place by a slight pressure; and a brush of charpy is carried into the interior, to wipe the surface of the neck, which is usually covered with mucosities, even in the sound state.

All these researches must be made in a clear light, to see, thoroughly, the cavity of the speculum, and the neck of the uterus. The patient is, for this purpose, placed before a window, or a candle is used." 47.

The following are enumerated as the states which contra-indicate the employment of the speculum.

"The presence of the hymen; and the extreme narrowness of the vagina in virgins; the rigidity which its orifice acquires in some old women; the excessive sensibility of the parts, in some cases, which may go the length of determining nervous accidents, by the slightest contact; painful and deep ulcerations of the vagina, and of the womb, are so many contra-indications. Obstacles, sometimes, occur in the cavity of the vagina, which is divided by membranous partitions. M. Lisfranc once met, at an inch from the os tincæ, a circular membrane, pierced with a central orifice, which opposed an obstacle at once to the touch, and to the speculum. The same practitioner met with a kind of *stricture* of the vagina, towards its superior third. The vagina may be, also, the seat of tumours, which prevent the speculum from penetrating.

The state of the womb itself interdicts, sometimes, the use of the speculum. Bleeding, and deep ulcerations of the neck; its enormous development; the fungosities which arise from its surface, prevent, and render even useless this means of exploration. The inflammatory engorgement of the neck in a very acute stage; that of the body of the organ, and the extreme sensibility with which this last state is accompanied; a sensibility which extends to the suspensory ligaments, and which the slightest movement increases." 48.

Of course the speculum should not be introduced at the catamenial period, nor during menorrhagia.

We think we need say no more on the employment of this instrument. In the present constitution of society in *this* country, we are confident that it will not be generally submitted to by females in the respectable classes. The cases in which it is absolutely necessary are not numerous, and in none but those would a physician or surgeon venture to propose its application to an English lady. We are not now reasoning the case in any way. We are simply stating what we believe to be the fact. Whether Dr. Balbirnie's persuasions will alter the feelings of females on this subject, or whether, with the changes occurring in society, they will eventually alter themselves, is a question which we do not pretend to discuss. We speak only of things as they exist.

But, in hospitals, and especially in Lock Hospitals, the speculum may and should be more extensively employed. It is a means of obtaining additional exactness in our acquaintance with disease, and that is sufficient to induce a philosophical physician or surgeon to employ it, whenever the opportunity presents itself.

The succeeding chapters are eleven in number. They are occupied successively with the consideration of the following subjects:—The Causes of the Organic Diseases of the Womb—The Symptoms of those Maladies—The Disorders of Menstruation—Uterine Hæmorrhages; Discharges of Blood that are not Menstrual, and not the Result of Child-birth—The Mucous Discharges from the Vagina, denominated, when simple, Leucorrhœa; when specific, Gonorrhœa—The Ulcerations of the Neck of the Womb—The Simple Hypertrophy, Chronic Inflammation, and Engorgement of the Womb; and the Simple and Scirrhous Induration—Cancer of the Womb—Polypi, and Fibrous Tumors of the Womb—Medical Treatment of the Organic Diseases of the Womb—Surgical Treatment.

Such is the Bill of Fare. A slight acquaintance with the obstetrical cuisine, informs us that there are in it several French dishes. Much has latterly been done in France to give precision, by dissections, to our knowledge of uterine diseases, and it must be owned that some of the older practitioners in this country are sadly in want of this sort of information. Our accoucheurs, till lately, were at a stand-still in pathology, and were far too busy on the surface of their craft to attend to the interior. They became excellent midwives, but *midwifery* was still their art. Such men as Dr. Robert Lee, Dr. Merriman, Dr. Locock, and others, have raised that art into a practical science, and have redeemed the characters of English accoucheurs. Perhaps, therefore, a survey of some of the chapters of this book, may enable us to pick up some useful knowledge for our older, or less experienced readers.

The second chapter is on the Causes of the Organic Diseases of the Womb. There is nothing novel nor indeed striking in Dr. Balbirnie's observations. He enumerates as causes of uterine maladies, the congestions it is liable to, in menstruation, in child-birth, in venereal congress, at the cessation of the catamenia: and he dwells, properly enough, on the bad effects of venereal indulgence, and on the influence of moral impressions. The only points to which we think it necessary to advert are these.

1. Organic alterations of the womb seldom occur before the age of puberty. But M. Lisfranc has seen patients, who dated the origin of their complaints to a period prior to that epoch; and M. Carron du Villards has met with a polypus, with engorgement of the body of the womb, in a child of seven years old.

2. Dr. Balbirnie alludes to a cause of disease of the neck of the uterus, which obtains, perhaps, more frequently than is imagined. It is observed, he remarks, in very young women, that the affection usually occupies the body of the womb, while, in those living in sexual communication, it is the neck that is the seat of the disease. Here, there is a direct action—a positive contusion of the neck—the result of venereal excesses, or of the disproportion of the organs. It is common in the newly-married.

3. He gives a hint with respect to scanty menstruation, which is worth attending to. Corpulent women, he says, with scanty menstruation is so common a circumstance as hardly to constitute a pathological state; but if the patient is thin and meagre, the reverse of plethoric, and with but a scanty menstruation, we may be almost certain that there is something anormal as regards the state of the uterus. It is difficult, perhaps, to determine the exact relation between the corpulency and the scantiness of menstruation; the relation, we mean, of cause and effect. When menstruation ceases women usually become corpulent, and so they do when it diminishes. Thin women usually menstruate too much, and, probably, are thin because they do so. When they remain thin with slight menstrual discharge they had best look to it.

4. Why does cancer attack the womb? Why does it attack the breast, the tongue, the testicle, the bones? The reason is equally difficult in all cases. No doubt there is a predisposition to the morbid change in some persons, which is little more than saying that some persons get it because they get it. That predisposition is, probably, connected with some subtle peculiarity of structure, for it is seen to prevail in families.

Bayle* mentions having seen, in a family composed of five individuals, a cancer of the breast in one, of the face in another, and of the stomach in a third. A woman, who died of an ulcer of the womb, had two sisters, one of whom died of a cancer of the breast; the other, then living, had a cancerous tumor of the neck. In another family, the father had a cancer of the tongue, and the son a *noli me tangere* of the face. M. Lisfranc mentions a family composed of seven daughters, the mother of whom fell a victim to cancer of the womb, and in which it has proved fatal to several of the others, and some have been attacked with uterine disease in a less or greater degree.

The third chapter is on the symptoms of the organic diseases of the womb. There is in it much "more noise than wool"—something to read, but little to remember. The author retails the sentiments of French authors, especially of M. Lisfranc, the droppings of whose cliniques into his pages form a considerable portion of their contents. Our object is not criticism, or there would be little difficulty in finding matter for it; but the selection of what may afford our readers an useful hint. The only passage that will answer our purpose is the following.

"But the attention of practitioners deserves to be called to the subject of *prolapsus*; for a prevailing error has run through all the books of practice as regards this point. Here the *descent* is all that is looked to; and without taking into consideration the pathological state of the organ which has produced it, the application of a pessary is counselled: and it is not to be wondered at, if, instead of seeing the symptoms disappear, they become only aggravated by the presence of a foreign body, which adds to the intensity of the original cause—the irritation and engorgement of the womb. This descent is attributed by accoucheurs to a primitive weakness and relaxation of the ligaments of the womb. But this is purely hypothetical and gratuitous. We cannot appreciate this primitive weakness and relaxation of the ligaments. We know not what it is, nor have all the books on midwifery demonstrated it. But here is a cause, plain and palpable, that appeals to the reason of every man. The descent is, in fact, a uniform and universal result of the engorgement—augmentation of the bulk of the womb—a mere consequence of the law of gravity, and with which a primitive weakness of the ligaments, which is extremely forced and conjectural, has very little to do. We do not deny, indeed, but that in some cases the ligaments of the womb may be occasionally weakened, as accoucheurs contend, to account for this symptom. But such cases are extremely rare: so much so, that M. Lisfranc and others, who have had the greatest practice in these diseases, affirm never yet to have seen a case attributable to this cause." 68.

Our author goes on to observe, that ulcerations on the neck of the uterus are a frequent cause of the engorged condition, which, as well as the prolapsus, usually disappears when the ulcerations are healed. He cautions the practitioner against the recommendation of coition, for prolapsus uteri, a recommendation offered with the view of inducing pregnancy.

There is no doubt much sound sense in the preceding observations, and much truth in the denunciation of the abuse of pessaries. But there can be little question that Dr. Balbirnie goes rather too far when he repudiates, as he

---

* Traité des Maladies Cancereuses.

seems to do, the notion of prolapsus occurring independently of actual uterine alteration. Such cases are not so common as the world believes, but they are more common than Dr. Balbirnie implies.

The two succeeding chapters—on the Disorders of Menstruation and on Uterine Hæmorrhages, are avowedly little more than transcripts of M. Lisfranc's lectures. An easy mode this of writing a book. The French Professor combats errors very prevalent in France, which the verbatim translator would have us think, or thinks himself, are equally prevalent in England. But he is deceived, and pages of inapplicable verbiage might be usefully put into Mr. Bramah's press, and reduced to an appropriate—a trivial bulk. The points deserving notice in these same chapters are the following.

1. Dr. Balbirnie informs us that amenorrhœa and dysmenorrhœa are not, as is commonly supposed by practitioners, essential diseases, but depend, in the great majority of cases, on an engorged state of the uterus. For a knowledge of this, he says, we are indebted to that speculum which he ever lauds. Now amenorrhœa and dysmenorrhœa are, on the whole, affections of young unmarried females, and we may conceive the sort of delicacy which would lead us to use or to propose *such* an instrument in such patients. What practitioner ever dreams, in a case of this sort, of even hinting at "the touch?" There are few young ladies who do not, before marriage, suffer more or less from one of these affections, and few, we suppose, did our author's notions become fashionable, would fail to have had the speculum thrust into their vaginæ before they entered on the state of matrimony. Happy æra! fortunate generation! so philosophical as to discard those obsolete notions of propriety and decency, which proved in our forefathers, as well as in our fore-mothers, so imperfect a civilization. The recommendations of Ovid to the young and erring bride will no longer be required, to enable her to hide her crime. The speculum will henceforth afford an apology for virgins without virginity, and for brides whose passions have been troublesome.

It is difficult to conceive where Dr. Balbirnie ascertained the nature of the professional information of his countrymen. He every where attributes to them so strange and so utter a lack of information, that we should doubt his intimate, certainly his personal acquaintance with the best practitioners. When he knows them better, he will find that they do not either view or treat amenorrhœa or dysmenorrhœa, in the unphilosophical and empirical manner he supposes.

But we tax the speculum, used indiscriminately as it appears to be, with the very fault its advocates profess it alone obviates—we tax it with occasioning empiricism. What should we think of a man, who settled all questions concerning the state of the leg by a glance at the great toe? The person who affects to determine, by a view of the os uteri, what the state of the whole uterus in all cases is, commits almost as great an absurdity. One would imagine that we possessed no valuable indications of the condition of the uterus in the general, and in particular symptoms, independently of what either sight or touch could offer. Such must be the conclusion that an inexperienced reader would draw from the constant tenor of Dr. Balbirnie's observations; yet that conclusion would be utterly preposterous. We do not say that the assistance of the speculum would not be both desirable and valuable, supposing there were no material objections to it. But we do say that, in the great majority of cases, both of amenorrhœa and dysmenorrhœa, we can do very well indeed without it, and that no man possessed of a sense of propriety would dream of recommending its employment.

In what Dr. Balbirnie says of the abuse of emmenagogues we coincide with him. The only point on which we differ from him, is in conceiving that the well informed do abuse those remedies. There is not a good physician in this metropolis, who, on a case of amenorrhœa or dysmenorrhœa presenting itself, would not carefully investigate the circumstances, to see what was wrong in the particular instance, and endeavour to set *that* right. Whether London physicians would do what follows is not quite so clear. We are presented with a *young* patient of feeble health, of soft flabby tissue, in whom the *presumed* age of the menses has arrived, and yet the *prodromes* of it are wanting.* For such a patient, in addition to baths and exercises, local means also are to be employed.

"Stimulant foot baths, *aromatic fumigations,* small lavemens, *very hot,* warm injections into the vagina, if the hymen permits it: in like manner, local hot baths established in the vagina, by putting the pelvis in an elevated position; emollient hip baths; warm cataplasms around the pelvis and to the pudendum; *dry cupping-glasses,* or scarifications in *the vicinity;* blistering, or sinapisms; the application of leeches in small number to the ankles, to the legs, to the internal and upper part of the thighs, rarely to the vulve; small blood-lettings from the foot; partial baths, in which the water is to the height of the knees, experience having proved to M. Lisfranc, that the baths in which the feet are merely dipped, are more injurious than useful." 83.

There is a pretty category. Imagine the assiduity of the medical attendants on this young lady. In rapid succession, or perhaps at once, her doctor, ascertaining that her hymen will permit it, syringes a warm injection into her vagina—behind, her nurse is anxiously throwing up a lavement very hot—the cupper has succeeded in fixing some dry cups on her pudendum—whilst the mother is invoking the unwilling uterus with an aromatic fumigation. Attacked in front, in rear, upon the flank, can the enemy resist? The blushing girl, but recently instructed in the alterations of puberty, discovers with astonishment the importance of parts of which she scarcely thought before. Incense is offered—oblations are paid—to that animal in animale, with which she is made so particularly acquainted, and her sense of modesty must be infinitely heightened by the delicacy of the various means that are resorted to. It at first seems odd how a local hot bath can be established in the vagina, by putting the pelvis in an *elevated* position. We would suggest a method more effectual, and quite as efficacious as any that we read of—let the interior of the vagina communicate, by means of a leathern tube and pipe, with a reservoir of warm water at some height. This is the mode of filling baths adopted in hospitals, and would answer excellently in supplying the vagina. Or, a small apparatus, like a fire engine, might be made constantly to play into it. Seriously, who does not see that

* Our author has imported a whole vocabulary. Scarce a page that has not its half dozen of Gallicisms. The construction of the sentences is neither French nor English, but something between both—a singular lingo.

measures of this description are not at all adapted for respectable young females in this country?

2. We may extract the following observations of M. Lisfranc.

"He has met with women who did not menstruate but every five or six months, or every three, four, or even six years. Sometimes they are habitually suffering, and then the indication is the same as for those who do not menstruate at all; at other times they apparently enjoy good health. But we must be upon our guard, that this deceitful calm does not seem to disguise some grave affliction, which will be revealed at a later period: a disease of the heart; a latent peritonitis, or some chronic alteration of the pulmonary organs. M. Lisfranc has known three young women who had not had children, and who menstruated but rarely: all the three died, one of them at the age of twenty-one, of disease of the heart; the others at the ages of nineteen and twenty-four, of tubercles in the lungs. The defect of menstruation, was it the cause or the effect of these accidents? He inclined to the former opinion; because, in the first instance, these women offered no symptoms of the diseases they died of, and which were only consecutive. Do we not see every day, the establishment of the menses dissipate grave disorders affecting other organs? From these considerations, M. Lisfranc thinks it useful to practise from time to time a small revulsive bleeding from the arm, and to prescribe an appropriate regimen. Frequently, when the amenorrhœa has been conjoined with some grave affection of another organ, the appearance of the menses has been seen to revulse powerfully, and to dissipate this last disease, or at least to arrest its progress. The professor has followed the principle laid down, with great advantage, upon many women. Among others, one aged thirty-six years who has not menstruated for six years, finds herself well from this precaution. To do nothing in such a circumstance would be at least a fault." 89.

There is no doubt that amenorrhœa is both the cause and consequence of visceral disease. To ascertain which it is in a given case, it is necessary that the particulars of that case should be investigated. One word with regard to revulsive bleedings, so fashionable in France. They are not practised to nearly the same extent in this country, nor need they be, for here the value of purgatives is much better understood. We are not here preferring the one or the other, but simply stating the fact—that purgatives, with our females, are the substitute for leechings and bleedings abroad. If we are to judge by Dr. Balbirnie's book, itself an echo of Continental practice, we should say that, in all that concerns remedies, we are much in advance in these maladies, as in most others, of our foreign neighbours.

"Stormy menstruation" next attracts the attention of our author, or rather of M. Lisfranc; but we see no inducement to launch ourselves upon its troubled waters.

3. The only remaining point, indeed, in this chapter requiring allusion, is embodied in an observation on the cessation of the catamenia. We are not, says our author, to allow ourselves to be led away by the opinion long time taught, that affections of the womb are more frequent at this time than at any other. It is from twenty to thirty-five years of age that the sexual organs are most exercised; it is between these two ages that their diseases are, also, most frequent. All our observation in the hospitals is confirmatory of this fact. It is not generally conceived that affections of the womb are, in the gross, more frequent about this epoch, but that cancerous alterations are so. And there is every reason to believe that this is true. With this reservation, Dr. Balbirnie's remark deserves attention.

The fifth chapter, on Uterine Hæmorrhages—Discharges of Blood that are not Menstrual, and not the result of Child-birth—may present one or two topics to detain us.

Uterine hæmorrhage, dependent on diseases of the womb, is no doubt frequently mistaken by ill-informed persons for the menstrual flux. A dangerous error, and one that is not likely to be committed after a moderately careful investigation of the circumstances. We need not stop to point out the distinctions between such hæmorrhage, and the catamenia. They are simple, and must be familiar.

But, when hæmorrhage of this description has lasted for some years, and become a habit, its sudden suppression may be supposed to be, and is dangerous. The following cases, related by M. Lisfranc, are to the point.

"One of M. Lisfranc's patients, aged twenty-eight, having never had a child, was subject, during twelve years, to discharges from the uterus, appearing regularly before the menses. The first time that he wished to suppress the hæmorrhage, she was attacked with peritonitis, as the consequence: on the second attempt, notwithstanding preparatory bleedings, peripneumony declared itself: the third time, it was an inflammation of the meningeal membrane. All these accidents yielded, as if by enchantment, to the application of leeches to the pudendum.

Another of his patients suffered, during eight years, a similar discharge, kept up by an engorgement of the womb. A revulsive bleeding from the arm arrested the hæmorrhage, but there supervened cephalalgia, or some other affection, which was only dissipated on the return of the flux.

Another young patient of his, having tubercles in her lungs, experienced abundant sanguineous discharges. 'I was here upon my guard (says the Professor), not to suppress them entirely; I only endeavoured to moderate them; and, as an aggravation of the disease of the breast threatened, I took care to recal the blood towards the womb. By this simple, but rational conduct, I had prolonged the existence of this young patient for three years, during which the phthisis seemed to be rendered stationary. She went into the country: the new physician, who had the charge of her, had no other anxiety but to suppress this uterine hæmorrhage, to which he attributed the feeble health of his patient: in a few months she was conducted to the tomb.'* " 101.

The causes of uterine hæmorrhage are various, and we need not enumerate them. It is the practitioner's business to ascertain their nature in particular cases. But our author looks on the hæmorrhage in a triple view. For, first, either the principal affection is curable, and the discharge may be suppressed without danger; or, secondly, the hæmorrhage is connected with a grave affection of some other organ, which its suppression would inevitably aggravate; or, lastly, it depends on an incurable uterine affection. The fact is, that the general principles which regulate our treatment of the hæmorrhages, must regulate us in the instance of the uterus, and to these general principles, we see in the pages now before us no important addition.

The sixth chapter is on leucorrhœa and on gonorrhœa. It offers even more decidedly than those before it, an example of the hop-skip-and-jump style of writing. Every thing is positive—the representations of disease are tableaux—the speculum achieves miracles—the treatment is slap-dash.

---

* Leçons de Clinique.

It is eminently the chapter of one who takes what others have said for his creed, and gives, at second-hand, Continental professorial pathology.

We are told, for example, that "the general application of the speculum in France, within the last few years, to the diagnostic and treatment of the diseases of the womb, has thrown an entirely new light on the pathology of this very common and very inconvenient infirmity in woman, as well as given an almost infallible certainty to its therapeutic."

That routine practitioners are apt to look on leucorrhœa with a very empirical eye, it would be improper to deny; but that well-informed men commit such monstrous and absurd mistakes, we are by no means disposed to admit. They connect the discharge with the state of the health and of the part, and suit their general and local remedies to both. What good physician, what practical surgeon, when a case of leucorrhœa is presented to him, does not try to ascertain whether it depends on a chronic inflammation of the uterus, or simply on an atonic condition of the mucous membrane of the vagina, and of the vaginal and uterine follicles? Our author would have us believe that general symptoms do not enable us to form an opinion. As practical men, we contend that, in the great majority of instances, they do, and it is only when the disease resists the means which those general symptoms indicate, that we are called upon, in most instances, to request the opportunity of instituting a more particular examination. But let us listen to Dr. Balbirnie.

"What is the pathological condition of the organ which furnishes this discharge, as demonstrated by the speculum? *Chronic inflammation, engorgement, ulcerations and erosions of the mucous membrane of the neck of the womb, and of its orifice,* in the infinite majority of cases. The great seat and source of the discharge, we have already mentioned to be *in the mucous follicles of the interior of the neck; and rationally and effectually to cure the disease, our remedial applications must be made to bear directly upon that point; which cannot be done without the speculum.* When the thick mucosities which frequently cover the os tincæ, and obstruct its orifice, are cleared away, there is, in the simplest cases, an erythematous blush, of greater or less extent, around the orifice, and sometimes invading the whole neck. Upon the greatest number exist superficial ulcerations of one or both lips, and very frequently running in the orifice of the neck. The deep red tint of the affected parts forms a lively contrast with the white greyish colour of the surrounding sound portion. Sometimes the whole surface appears as if covered with granulations, which are nothing else than the enlarged mucous follicles of the membrane. In the acute *vaginitis* this granular appearance is very remarkable. Sometimes there is a true irruption of *herpes phlictenoides* on the os tincæ. When the disease is of considerable duration, the ulceration extends profoundly into the neck and cavity of the uterus. The parts which are the seat of these lesions offer, in the greatest number of cases, an augmentation of volume. Sometimes the anterior lip is hypertrophied; sometimes the posterior, or the whole neck: it is sometimes enormously enlarged; and we have seen ulcers on it of two inches to two and a half inches in extent, covered often with a glutinous secretion, of a greyish yellow colour, and resembling a wound attacked with hospital gangrene. The displacement and descent of the womb almost universally accompany this state; as also anteversion, retroversion, and the lateral inclinations." 110.

Who does not see in this a caricature, rather than a plain and faithful picture? The great majority of females above twenty years of age labour, more or less, under leucorrhœa. Yet in how small a proportion do serious pathological changes exist: in how large a number, is the essential condition a passive affection of the mucous membrane of the vagina and the uterus. Such representations as the one we have extracted are, however, useful. Their very extravagance makes them striking, and arrests the attention of careless and indolent persons. We repeat that we agree with Dr. Balbirnie in lamenting that too many entertain most empirical notions of the nature of leucorrhœa, and, perhaps, they may be awakened by such a blast as the one Dr. Balbirnie has blown, to the necessity of keeping their eyes open.

We may record the treatment pursued in France, which our author reports and recommends.

"Here the *local* treatment is the primary indication, and that by which alone we obtain a durable success. If it be ulcerations of the neck of the womb which have produced and keep up the disease, the cicatrisation of these ulcers is almost always followed by a notable diminution of the engorged tissues, and of the flux. *Cauterisation* is the remedy, *par excellence*, for these. This is performed generally once a week, with the nitrate of silver, or a solution of the nitrate acid of mercury."

"When the disease is very acute, the treatment is entirely antiphlogistic: bleedings from the arm, general baths, cold emollient injections, lavemens, and strict regimen. In other cases, the cauterisations *alone* generally suffice; and, often after a few repetitions of it, the pains of the loins, which were habitual, begin already to disappear—the discharge diminishes—the sleep, which had been troubled, becomes better—the appetite returns, and the disease points already to a favourable termination." 115.

We have usually more strings to our bow than we see here, and much may be done by great attention to the health, and by the medicines which correct secretions. But it is not our purpose to enter on such considerations.

"Where the discharge has its origin in an inflammation of the mucous membrane of the vagina, the solid cauterisation performed rapidly with the nitrate of silver, or astringent injections, with a pledget of charpie, imbibed in a concentrated solution of acetate of lead, renewed once or twice a day, and left applied to the neck of the womb, as practised by M. Ricord, is followed by invariable success.

When the discharge comes from the interior of the uterus, it is always glairy and transparent, like the white of egg; injections into the cavity of a solution of the acetate of lead, or of a very weak solution of the nitrate of silver, repeated every day, dries up in a short time the source of this very annoying infirmity." 116.

All is couleur de rose with our author. The speculum finds the disease—the caustic cures it. So may it be.

When the lining membrane of the uterus is the seat of "catarrhal inflammation," it becomes swollen and puffy—the organ is plugged up with thick and viscid mucus—sterility is the result—and serious organic alterations may be induced. For this M. Melier has proposed injections into the uterine cavity. After the cure of leucorrhœa, injections of cold water into the vagina should be habitually employed.

Ulcerations of the neck of the womb occupy the seventh chapter. Such ulcerations Dr. Balbirnie affirms to be the *most common of all* the affections of which the neck of the uterus is the seat. They may be so, but so far as our small experience with the speculum and without it has gone, they have not been so. Chronic inflammation of the cervix uteri has appeared to us to be infinitely more frequent.

The ulcerations may be superficial; or deep-eating ulcers, with hard and elevated edges; or a granulated surface like a blistered one; or the ulcer may have the form of a deep long fissure, seated on one or on both the lips of the os tincæ, and frequently running into the orifice of the uterus. The posterior lip of the os tincæ is the most frequent seat, and the orifice of the womb is almost always implicated in the ulcerations which are a little extensive.

The most common causes are contusions and lacerations of the os tincæ in difficult child-birth; the use of instruments to aid delivery; abortions; the wearing of pessaries; excess of venery; and the disproportionate size of the organs; causes, in short, that tend to keep up a chronic irritation and inflammation of the neck of the womb.

Our author adopts M. Lisfranc's classification of the ulcerations, with a very slight modification. It is the following.

1. The mere erythematous state of the os tincæ, with or without a phlictenular eruption.

2. Simple benign ulcerations.

3. Venereal ulcerations; *the true Hunterian chancre* seated on the neck of the womb.

4. Scrofulous ulcerations.

Lastly. Cancerous ulcerations.

1. In the simple erythematous state of the os tincæ, the redness is more or less extensive in surface, occupying either the whole of the os tincæ, or only circumscribed spots here and there; and it is generally accompanied with some engorgement and vascularity of the tissues. The mucous membrane is often thick and velvety to the feel, and sometimes bleeds with great facility.

There is frequently observed a multitude of small granulations, or minute miliary vesicles, distinct or confluent, which afterwards break, and become superficial ulcerations.

There may or may not be uneasiness with this state. When it results from acute gonorrhœa there usually is some.

2. Of the simple ulcerations we need say nothing. Their appearance may be readily imagined. They may occupy both lips of the os tincæ indifferently; most frequently, however, the posterior; or they may be seated on the edges of the orifice, and extend even into the cavity of the uterus, which is most commonly the case. If the mucous membrane be slightly swollen, inflamed, and œdematous, the ulceration appears deeper than it really is.

3. We will say nothing of venereal ulcerations of the cervix. We noticed three years ago a paper of M. Ricord's on this subject. We do not think that these ulcerations are by any means so common among the prostitutes in this country as that paper would lead us to suppose.

4. On scrofulous ulceration we must make the following extract.

"According to M. Lisfranc, the scrofulous ulcer is the consequence of the softening and opening of tubercles, and are preceded by a fluctuating tumour. Its opening is at first straight and fistulous—then it enlarges insensibly: the bottom of the kyste is of a pale, wan, grayish tint, with ragged and thin edges, giving issue to a matter of a disagreeable smell, but different from that of cancer. Frequently there co-exists an engorgement of the neck and body of the uterus. M. Lisfranc has met with about a dozen of these cases, in which the constitution of the patient, and the issue of the characteristic curdy matter (analogous to that of a suppurated cervical ganglion) left little doubt as to the diagnostic. The knotty feeling of the tumour renders its distinction from the scirrhous state often difficult. But the characteristic lancinating pains are wanting. The issue of the contents, and the facility and promptitude of the cicatrisation, evince sufficiently its true nature." 142.

5. The cancerous ulcerations are treated of in subsequent chapters. Our author communicates nothing with respect to them that merits particular attention.

We may dismiss the eighth, ninth, and tenth chapters without ceremony. They are appropriated to hypertrophy, chronic inflammation, and engorgement of the womb—cancer of the womb—and polypi and fibrous tumors of it. The chapters in question contain little that could be usefully noticed here.

The eleventh chapter is on the medical treatment of the organic diseases of the womb, and as it affords us a good insight into the practice adopted in France, we may feel some interest in glancing at its contents. The items of treatment are separately considered in the following order.

1. *Bleeding.* M. Lisfranc constantly employs this as a revulsive. His method is, during the intervals of each menstrual period, to prescribe two or three small revulsive bleedings from the arm, of three or four ounces only, whenever the uterine congestion and pains are intense : for eight days before and after this evacuation he abstains from bleeding, lest it should interfere with it. MM. Duparcque and Tealier practise it a few days before the occurrence of the catamenia, in order to moderate the congestion they entail upon the uterus.

2. *Application of Leeches to the Neck of the Uterus.* There appears to be some difference of opinion with respect to the utility of this measure. It would appear that if *revulsive* bleedings are good, *attractive* leechings could hardly be so. Yet Dr. Balbirnie assures us that he has seen the latter beneficial. "The proof of the pudding is in the eating." The mode of applying the leeches is as follows. The speculum is introduced, and the neck of the uterus being embraced in its extremity, the mucosities are wiped away by a brush of charpy, or by an injection of tepid water. The leeches are introduced into the tube, pushed forward to the os tincæ, and kept applied by a pledget of lint, which is removed as soon as they attach. The bites are hardly felt: in ten minutes the leeches gorge themselves, and fall off. A few injections of tepid water are again made, and the speculum withdrawn. The greatest number of leeches that may be applied is twelve: six or eight are the usual quantity. The bites soon cicatrise, and rarely or never give birth to troublesome ulcerations.

3. *Baths.* Hip-baths have been recommended and are employed, almost as a matter of course in diseases of the womb. A great absurdity, in our author's opinion, nay, the very greatest possible folly. He reasons thus.

"For a headach, for example, we prescribe a foot-bath, to determine the blood towards the lower extremities; yet, for an affection of the pelvis, we do not fear to congestion the pelvis even! Is it not because they determine an afflux of blood towards the uterus, that practitioners prescribe them, when they wish to recal menstruation." Yet we may give the doctor a rap with his own cane. If hip-baths are so hurtful, because they draw the blood to the part, why should leeches, which do the same, be recommended by him? True, they deplete too, but do not warmth and moisture, which produce exhalation and secretion, deplete in some measure also? Has Dr. Balbirnie never seen fomentations, local hot-baths, applied to inflamed parts—to the surface affected with erysipelas, gout, or rheumatism—to the inflamed conjunctiva? By a parity of reasoning this should be absurd, yet it is found practically useful. The fact is, that hip-baths produce so much relaxation of the external parts and the vagina, that they unload the exhalent vessels. And, then, they are almost indispensable for cleanliness, in those organic diseases of the uterus, attended with offensive or profuse discharges. Dr. Balbirnie, however, affirms that the hip bath is in practice injurious, and he tells us that M. Lisfranc's method is to employ the general warm-bath, and to make the patient remain in it for at least two hours and a half, or three hours. This is to be repeated morning and evening on alternate days. Six hours a day in a warm bath is pretty well, and would be convenient enough in private practice.

4. *Rest* of body and of mind is obviously called for.

5. *Diet* need not detain us.

6. *Cauterisation.* This is extensively practised for ulcerations of the cervix.

"It is employed with two objects:—1. In the simple ulcers of the neck of the womb, on the principle laid down by Alibert in the case of cutaneous cancers—to the extent merely of altering the vitality of the tissues, and, by that means, hastening the cicatrisation. 2. In the cancerous ulcers, for the purpose of destroying the subjacent tissues, as well as repressing the excrescences. For the former, a gentle touching with the solid nitrate of silver, perhaps, answers best, and is constantly used with great success in the extensive practice of M. Ricord. M. Lisfranc, and others, prefer the nitrate acid of mercury, made by dissolving one part of the nitrate of mercury crystallised, in eight parts of nitric acid. This is diluted in twelve parts of water, and applied, by means of a fine brush of charpy, like a small camel-hair pencil—the speculum being previously introduced, the os tincæ embraced, and the mucosities wiped away; an injection of cold water is used by these practitioners before withdrawing the instrument. When the destruction of a cancerous ulcer, with large bleeding vegetations, is in question, the preference is given to the nitrate acid of mercury pure, as it penetrates and corrodes the tissues more profoundly. The extent and intensity of the cauterisation is always regulated by that of the ulcer. These cauterisations do not last two or three seconds at most, and are absolutely without pain in the immense majority of cases. By means of successive cauterisations, the whole of the neck of the uterus, and part of its body, may be destroyed, without danger or inconvenience to the patient; but, on the contrary, often prolonging her existence for several years. We have seen the cauterisation employed in some hundreds of cases, and there never has resulted the slightest accident worthy of note." 290.

We will not discuss the principle of treating cancerous ulcers or the cervix by caustics. We would only observe that, as they notoriously fail upon the surface of the body, they are not very likely to answer in the interior. The cauterizations are generally renewed every eight days—after each, a bath is prescribed—and daily injections are employed in the interval. Of course the menstrual periods are avoided. For the simple ulcerations, M. Ricord always employs a pledget of charpy imbibed in a solution of the acetate of lead, or of the *vin aromatique* of the French codex, and applied by means of the speculum to the os tincæ, immediately after the cauterization. Other caustic and stimulating applications have at various times, and by various persons, been employed. They scarcely deserve mention. One of the latest is the chloruret of platinum, patronised by M. Recamier.

7. *Injections* are of constant use. The patient should be so placed, that the fluid may tend by its gravity towards the uterus. M. Ricord gives the preference to the permanent application of liquids imbibed in charpy, and introduced by means of the speculum, and allowed to remain applied to the os tincæ twelve or twenty-four hours, according to the abundance of the secretion.

On revulsives, narcotics, irrigations, lavements, compression, iodine, tartar-emetic, ergot of rye, acetate of ammonia, acetate of lead, hemlock, we may advantageously be silent.

Nor need we open the discussion on the propriety of amputating the neck of the uterus. Whether that operation may not be in some cases practised with advantage, is one question—whether it has or has not been grossly abused, is another. We entertain doubts on neither. And if any one thing is more certain than another, it is this—that in the first place it will not cure true cancer of the uterus—and, in the second, that cases have been publicly reported, of cancer cured by it, when either there was not cancer at all: or when, being cancer, the patient has been finally destroyed by it. What, for example, shall we say of such gross want of faith as is exhibited in the following anecdote, published by Dr. Krimer, of Aix-la-Chapelle, and copied by Dr. Balbirnie:—

"Madame de C., aged forty years, had all the vaginal part of the uterus in a carcinomatous state. A practitioner made the re-section of it, in the presence of a great number of spectators. At the end of fifteen days, he declared the patient cured, and presented her, as such to a medical society. Her improved state continued during two months; at the end of this period, a leucorrhea commenced, and became more and more abundant, and more and more fetid. The lady was sent to the Spa-waters, and there was seized with several abundant menorrhages. She removed to Aix-la-Chapelle, seven months after being operated on at Paris. The touch recognized ulcerated vegetations, half the size of the fist, upon the neck of the uterus. Three months afterwards, she died at Ostend; and, nevertheless, she figures among the Cases completely cured by amputation." 308.

Of such a character, our readers may be assured, are the majority of cases of cancer of the uterus cured. Either, in ninety-nine of the hundred, there was no cancer or no cure. Of such a character, too, has been, in most cases, the good faith of those who have purchased a little present hollow reputation, at the expense of treachery to the profession, and calculating inhumanity to patients.

It is not necessary to pronounce a formal opinion on the work from which we part. There is in it much enthusiasm, which, rightly directed, and restrained by sound judgment, would be equally advantageous to the author and the public. The former is not undeserving of encouragement, yet a friendly critic would desire that the present volume were cancelled, and another, pruned of its redundancies and indiscretions, substituted, in a few years, in its place. The author has done well in directing attention to the employment of the speculum, but his advocacy has been indiscriminating and importunate, and, attributing too much to his favourite instrument, he is likely rather to damage himself than to benefit it.

